# The Unusual Role of Ribonuclease L in Innate Immunity

**DOI:** 10.1002/wrna.1878

**Published:** 2024-12-27

**Authors:** Agnes Karasik, Nicholas R. Guydosh

**Affiliations:** ^1^ Laboratory of Biochemistry and Genetics, National Institute of Diabetes and Digestive and Kidney Diseases National Institutes of Health Bethesda Maryland USA

**Keywords:** dsRNA response, innate immunity, mRNA decay, RNase L

## Abstract

Ribonuclease L is an endonuclease that is activated as part of the dsRNA‐driven innate immune response. Active RNase L cleaves pathogenic RNAs as a way to eliminate infections. However, there are additional and unexpected ways that RNase L causes changes in the host that promote an immune response and contribute to its role in host defense. Central to these unconventional mechanisms is the observation that RNase L also degrades the mRNA of the host. In turn, mRNA fragments that RNase L generates can be translated. This causes activation of a ribosome collision sensor that leads to downstream signaling and cell death. Additionally, the liberation of RNA binding proteins after RNA decay appears to affect gene expression. In this review, we discuss these and other recent advances that focus on novel and unusual ways RNase L contributes to innate immunity.

## Graphical abstract

RNase L's novel and unusual roles in host innate immune response and viral infection.

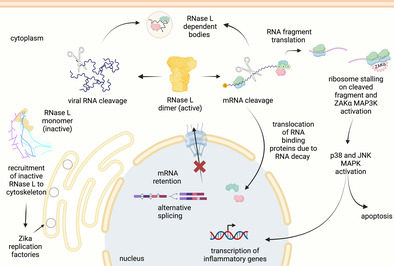

## 1 Introduction

**FIGURE 1:**
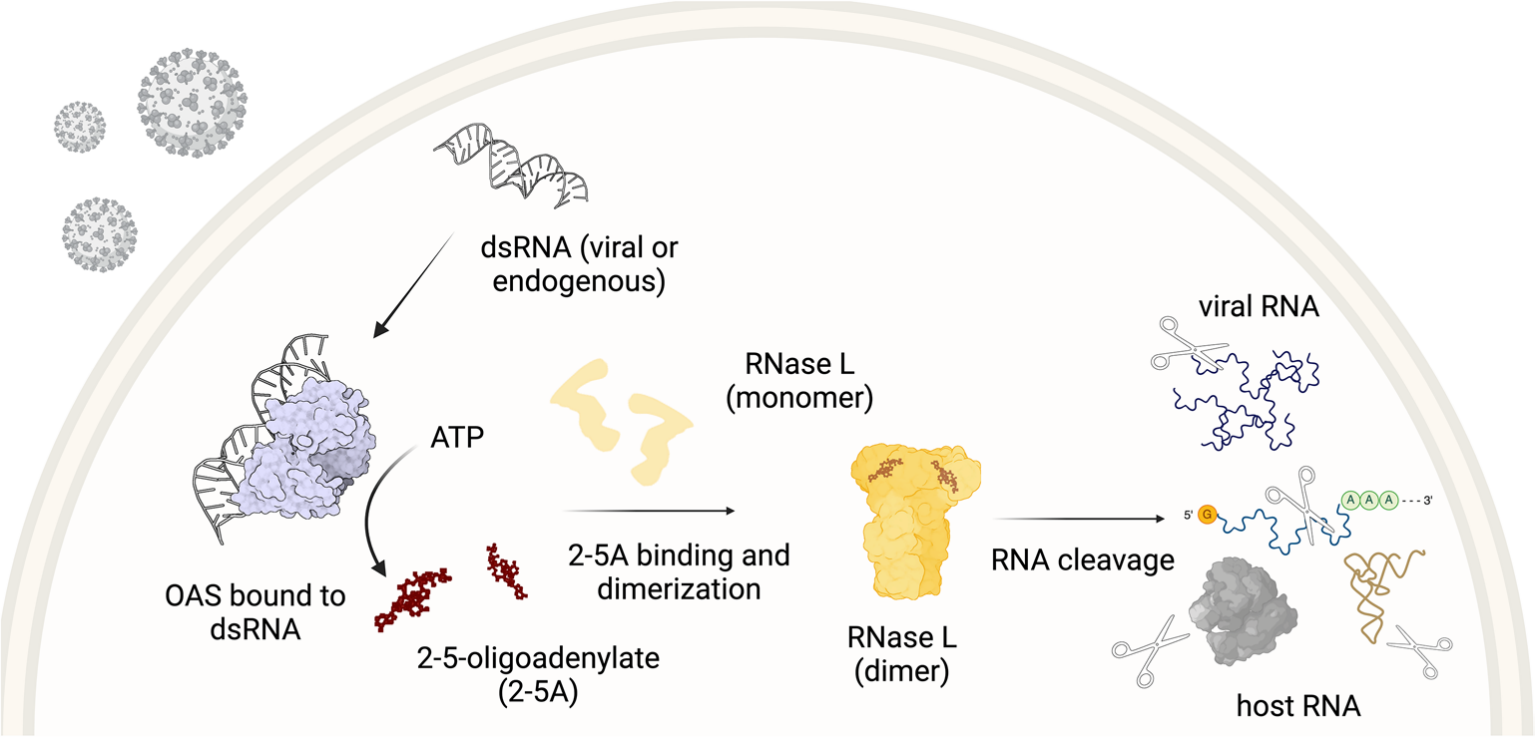
dsRNA activates RNase L and it then cleaves most species of RNA. dsRNA is generated in the cytoplasm due to viral infection or it can also be produced endogenously. OAS enzymes recognize dsRNA and, as a result, produce 2′‐5′ linked oligoadenylates (2–5A) from ATP. 2–5A binds the monomer RNase L, causing it to dimerize and activate its endonucleolytic activity. RNase L cleaves most RNAs in the cell, including viral and host RNAs (mRNA, tRNA and rRNA as part of the ribosome (grey) are depicted here).

Double‐stranded RNA (dsRNA) sensing is one of the critical ways the cell recognizes viral pathogens (Hartmann 2017). The Oligo Adenylate Synthases (OASs) are dsRNA sensors that constitute one of the major arms of the antiviral dsRNA response. They activate a host‐encoded antiviral endonuclease, Ribonuclease L (RNase L, “L” stands for latent), by producing a small molecule, 2‐5‐oligoadenylate (2–5A). Then, 2–5A binds to the monomeric form of RNase L and promotes its dimerization and activation. RNase L is an ~80 kDa protein that contains an ankyrin repeat domain, known for facilitating protein–protein interactions, that also harbors the 2–5A binding site (Han et al. 2014; Huang et al. 2014) (Figure 1). When RNase L becomes an active endonuclease, it cleaves most host and viral single‐stranded RNAs. RNase L is activated by a large subset of viruses, including SARS‐CoV‐2 and Zika, but also has functions in the absence of infection, including anticancer and immune regulatory properties (such as activation of inflammation and inhibition of cell migration) (Banerjee et al. 2015; Casey et al. 2002; Lee et al. 2023; Li et al. 2021; Whelan et al. 2019). Because of these links to infectious disease, cancer, and autoimmune conditions, a better understanding of its function holds great potential for the development of novel therapies to treat these respective conditions.

**FIGURE 2:**
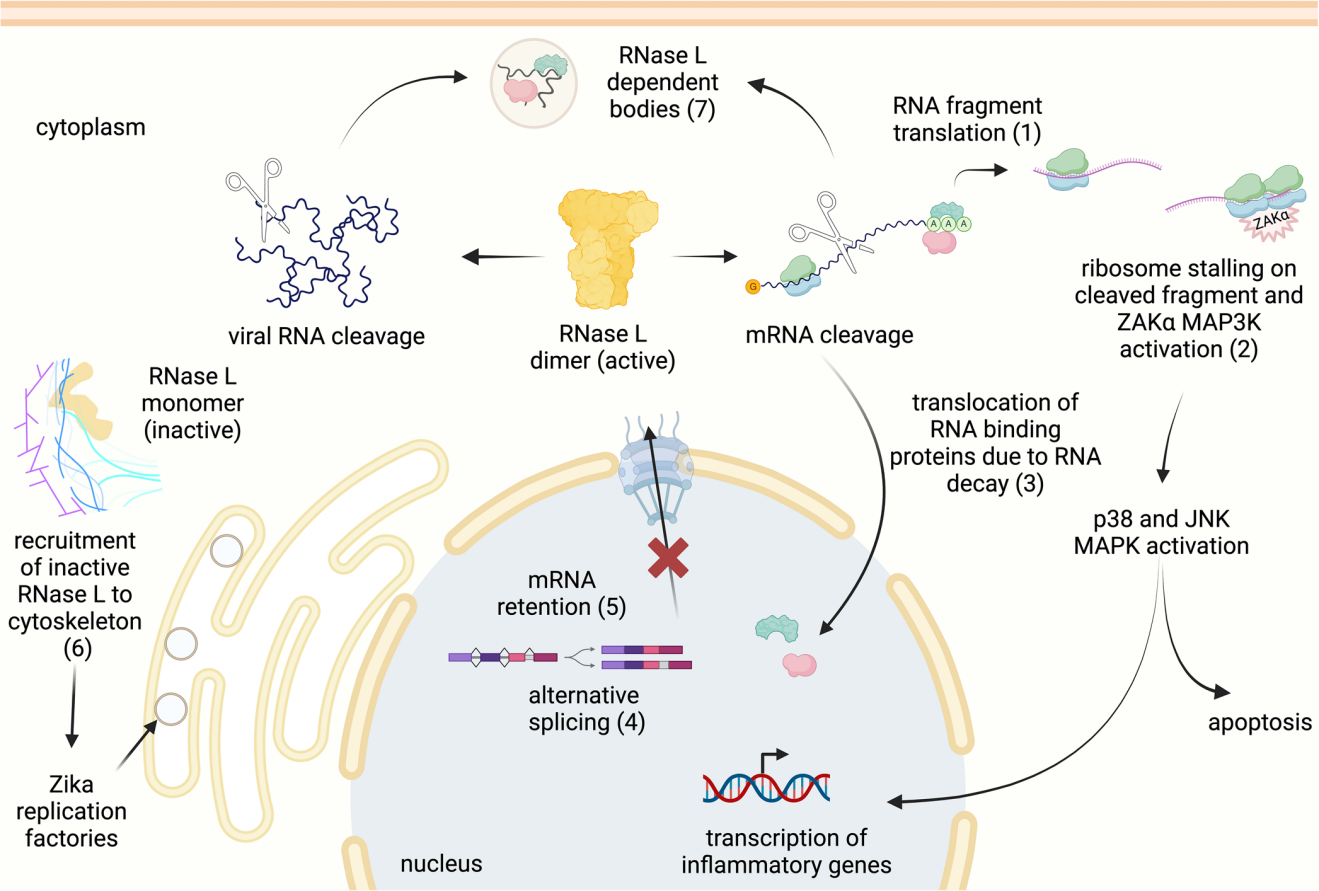
Recent studies have revealed novel roles for RNase L. Active RNase L cleaves host mRNAs into fragments that can be translated (1). This RNA fragment translation can induce ribosome stalling and/or collisions and recognition by a ribosome collision sensor, ZAKα, leads to an inflammatory response and apoptosis (2). The overall loss of mRNA in the cell increases the amount of free RNA binding proteins and results in their translocation to the nucleus (3). This is thought to be responsible of other observed effects of RNase L activation, such as effects on alternative splicing (4) and nuclear mRNA retention (5). Inactive RNase L was also shown to be recruited to the cytoskeleton to promote formation of Zika virus replication factories (6). Viral and host RNA cleavage results in formation of RNase‐L dependent bodies (7).

The outcome of RNase L‐mediated cleavage of viral RNAs is that it leads to their decay and limits viral replication. However, RNase L also degrades the RNA of the host, but the rationale for this process is less clear and thus we refer to this role as “unconventional” or “unusual.” Why would the cell degrade its own RNA? How does this activate the immune response and clear viral infection? One generally accepted view is that this widespread RNA cleavage promotes apoptosis of the infected cell, and thereby helps clear the virus from the organism (Castelli et al. 1997; Zhou et al. 1997). Intriguingly, some viral genomes encode nucleases that also carry out this role (Levene, Shrestha, and Gaglia 2021; Salgueiro et al. 2024). If RNA degradation benefits the host, why would a virus carry out a similar process? Here we address these questions by discussing recent findings about the unusual outcomes of RNase‐L driven changes to the host (Figure 2), including apoptosis. We also highlight how these discoveries reveal the importance of RNase L in human health and disease and could be further explored for developing therapeutic approaches.

## 2 How Does RNase L Select Its Substrates?

RNase L is known to cleave downstream of U bases (the consensus cleavage site is UN^N) (Han et al. 2014), with some preference shown in vitro for UU or UA sequence motifs (Floyd‐Smith, Slattery, and Lengyel 1981). Since a large subset of RNAs contain this motif, most of them are potential substrates of RNase L. Besides viral RNA, this includes host mRNAs, rRNAs as well as shorter RNAs, such as tRNAs and Y RNAs (Donovan et al. 2017). It is of a long‐standing interest in the field to understand how much preference RNase L exhibits for the selection of its targets and several models have been proposed in the literature (reviewed in (Brennan‐Laun et al. 2014)).

An early model predicts that RNAs with dsRNA regions could recruit OAS proteins, which have dsRNA binding activities, and this would generate 2–5A, the small molecule activator of RNase L, in the proximity of the double‐stranded feature of the RNA. The authors suggested this could result in localized activation of RNase L and thus preferential cleavage of regions containing dsRNA, particularly in viral RNA (Nilsen and Baglioni 1979). However, the ability of small molecules like 2–5A to diffuse rapidly make the proposed localization model difficult to envision without some role for subcellular compartmentalization of either the 2–5A or RNase L, such as viral factories. Moreover, a recent paper further countered the model (Harioudh et al. 2024). Harioudh et al. observed that the p46 isoform of OAS1 can bind host RNAs, including interferon‐β (*IFN* *‐β*), and this binding has a protective role and increases the half‐life of the mRNA rather than inducing its decay. This role of OAS1 was independent from its ability to synthesize 2–5A and thus suggests that other proteins could also make mRNA substrates of RNase L inaccessible to cleavage in the same way as OAS1. Intriguingly, another recent study showed that OAS3 can co‐localize with RNase L in dsRNA‐induced foci (Cusic and Burke 2024). This suggests that OAS3 could potentially also offer this sort of protection since these dsRNA foci appeared to be stable indicating that there is no RNA cleavage within the foci. The RNase L rapidly exchanged throughout the cytoplasm, consistent with a global, rather than foci‐specific, cleavage role.

Interestingly, some innate immune mRNAs can somewhat escape RNase‐L mediated decay (such as *IFN‐β*) due to low enrichment of these cleavage motifs (Burke et al. 2019; Rath et al. 2019), suggesting that selection by RNase L is based on nucleotide content (Rath et al. 2015). In addition, transcriptional upregulation of these genes also counteracts the effects of decay (Burke et al. 2019; Rath et al. 2019). Furthermore, it was observed that RNase L degrades host RNAs to a larger extent (nearly 100% in many cases, (Rath et al. 2019)) than SARS‐CoV‐2 RNA (~60% reduction (Burke et al. 2021b)). This indicates that host RNAs may be preferred over viral RNA in this case and therefore could point to the importance of RNase L's unconventional role in degrading the host's RNA. Further studies, such as RNA‐seq that is normalized to exogenous spike‐in RNAs in viral infected cells, would be interesting to further characterize how this preference changes as overall RNA levels drop in the cell during infection.

## 3 What Is the Role of Translation in RNase‐L Mediated Immunity?

### 3.1 Can RNase L Activity Be Modulated by Ribosomes?

Protein synthesis is important for innate immunity because it allows for the production of proteins needed for the interferon and inflammatory responses. In addition, translation was proposed to play an unusual role in modulating RNase L's endonuclease activity or targeting (reviewed in (Brennan‐Laun et al. 2014)). It was suggested that mRNAs that are bound by ribosomes are preferentially cleaved by RNase L. In support of this model, RNase L is found in the polysome fraction in the cell, indicating that it may associate with actively translating ribosomes and mRNAs (Salehzada et al. 1991). RNase L cleaves accessible loops of rRNAs and exhibits a well‐defined cleavage pattern in rRNA cleavage assays (Rath et al. 2019; Wreschner et al. 1981). However, ribosomes with rRNA cleaved by RNase L can still carry out translation comparable to that of uncleaved ribosomes (Rath et al. 2019). In addition, mRNA cleavage appears to precede rRNA cleavage (Brennan‐Laun et al. 2014; Nilsen, Maroney, and Baglioni 1982), suggesting that rRNAs are not targeted to eliminate their ability to translate and could instead signal a role in recruiting RNase L. Furthermore, cleavage rates of mRNAs by active RNase L follow Michaelis–Menten kinetics in reconstituted in vitro cleavage assays that lack components of the cytosol (Carroll et al. 1997), however, this is not true in cells (Rath et al. 2019). This suggests that there is an unidentified modulator of RNA cleavage in vivo. Based on all these observations, it is conceivable that ribosomes modulate RNase L's activity. However, another study (Burke et al. 2019) showed that release of ribosomes from mRNAs with puromycin had no effect on cleavage rates, and hence further work is needed.

Interestingly, specific translation factors were shown to directly bind to active RNase L (Bisbal et al. 2001; Le Roy et al. 2005, 2007; Nogimori et al. 2019) and these interactions could be important for modulating RNase L's activity or targeting. However, the exact functions of these interactions are not well‐studied and, in some cases, are controversial. For instance, two of these translation factors are eukaryotic polypeptide Release Factor 3 (eRF3) and an ABC protein family member, ABCE1, which play essential roles in translation termination and/or ribosome recycling at stop codons in cells (Young et al. 2015). Interestingly, ABCE1 is known to be a ribosome recycling factor but was first identified as a protein that could bind and inhibit RNase L (Bisbal et al. 2001). This interaction was proposed to be important for viral fitness since the lack of ABCE1 results in higher titers of virus during infection (Ramnani et al. 2021). In contrast, others showed that ABCE1 enhances, and does not inhibit, RNase L activity in HeLa cells (Nogimori et al. 2020). Thus, more study is needed to elucidate the role of ABCE1 and other translation factors in innate immunity and RNase L activation. RNase L was also reported to localize inside the mitochondria and directly interact with a mitochondrial translation initiation factor (IF2mt). This interaction was proposed to guide RNase L to mitochondrial transcripts, leading to their decay (Le Roy et al. 2007). Chloramphenicol, a drug that can halt protein synthesis in the mitochondria by preventing peptide bond formation, inhibited this IF2mt‐dependent activity of RNase L and stabilized mitochondrial transcripts. This result is therefore consistent with a role for the mitochondrial translational machinery in regulating RNase‐L driven RNA decay.

### 3.2 How Does Active RNase L Affect Translation?

During RNase L activation, ~90% of host mRNA is lost, with individual transcript loss varying from 60% to 99% (Rath et al. 2019). This small population of mRNA that remains retains the ability to be translated. How efficiently this mRNA pool is translated could affect the innate immune response during RNase L activation.

We investigated translation of this remaining pool of mRNAs by comparing data from RNA‐seq to ribosome profiling, a high‐throughput ribosome footprinting method (Ingolia et al. 2009). This offers a measure of translation efficiency (TE), or how well mRNAs are translated after RNase L activation (Karasik et al. 2024). While most TEs that were calculated for individual mRNAs were not affected by activation of RNase L, there were several notable exceptions. For instance, several proinflammatory mRNAs (e.g., *CXCL‐8*), which are transcriptionally upregulated, are further boosted in expression by increased translation efficiency when RNase L is active. We assume that the number of ribosomes during RNase L activation remains about the same while most mRNAs are degraded, potentially creating an excess of translational capacity. Transcripts with boosted TEs may take advantage of this excess of ribosomes during RNase L activation. Another possibility is that RNase L activation changes initiation factor availability and as a result these transcripts can recruit more initiation complexes. However, the exact mechanism of how RNase L activation changes these translation efficiencies remains to be elucidated. In contrast, transcripts that encode mucin proteins, such as *MUC5AC*, are also transcriptionally upregulated but are downregulated at the translational level, thus counteracting the transcriptional effect. Interestingly, active RNase L and a viral non‐specific endonuclease, nsp1 from SARS‐CoV‐2, induced the same effect for *IFN‐β* mRNA and it appeared that this is due to this mRNA being retained in the nucleus (Burke et al. 2021a, 2021b). Thus, it is plausible that translational inactivation during RNase L activation could also arise due to nuclear retention.

### 3.3 Endonucleolytic Cleavage by RNase L Induces Non‐canonical Translation

An intriguing finding to emerge from ribosome profiling data was that activation of RNase L increased the proportion of ribosomes in non‐coding regions of mRNAs (regions that are not translated in normal cells), including 5′UTRs, 3′UTRs, and out‐of‐frame regions of the coding sequence. Within these regions, we noted that ribosomes translated short “alternative” Open Reading Frames (altORFs) (Karasik et al. 2021). Earlier studies suggested that RNase L might lead to increased readthrough of stop codons by the translating ribosomes, resulting in 3′UTR translation (Le Roy et al. 2005). In contrast, our data suggested that the non‐canonical translation occurred throughout the transcript due to initiation on these ORFs and did not happen exclusively in the 3′UTR, excluding this earlier model. As a working model, we proposed that ribosomes initiate on cleaved fragments due to mass action driven by the large abundance of ribosomes in cells where most mRNAs had been lost (Figure 2). However, the exact mechanism that allows ribosomes to initiate on RNA fragments needs further work. In addition, it is not clear where mRNAs are fragmented by RNase L, and how long they remain stable in the cytoplasm before being degraded by exonucleases. Apart from altORF translation, this question is of interest since RNA fragments were reported to activate another branch of the dsRNA response (Malathi et al. 2007). However, detection of these fragments is technically challenging. Newer technologies, such as modified Nanopore sequencing, offer promising avenues for detection.

### 3.4 RNA Fragmentation Activates a Ribosome Collision Sensor, ZAKα

Since ribosomes translate fragmented mRNA, it is also important to ask whether this process leads to any other consequences that offer benefit to the cell. Ire1, an endonuclease and a key player of the Unfolded Protein Response (UPR), causes ribosomes to stall on fragmented mRNAs in fission yeast (Guydosh et al. 2017). These stalling events also lead to ribosome collisions (termed “disomes”) at these 3′ ends. Ribosome collisions occur when a ribosome stalls on the mRNA and blocks the way of the next ribosome, forming a “dimer” in the process (Meydan and Guydosh 2021). These findings therefore motivated studies that explored the possibility that activation of RNase L could also induce ribosome stalling, collision, and disome formation at the 3′ cleaved RNA fragments (Karasik et al. 2024; Xi et al. 2024).

RNase L activation was known to induce cell death through JNK (c‐Jun N‐terminal Kinase) MAP kinase activation (Li et al. 2004). While this pathway was originally termed the “Ribotoxic Stress Response” (RSR) because ribosome inhibitors could also induce it, the exact molecular mechanisms of how widespread RNA cleavage can lead to this signaling remained unknown. Interestingly, it was recently shown that ribosome collisions induce the RSR and result in JNK, as well as p38, MAPK activation (Vind et al. 2020; Wu et al. 2020). In particular, disome recognition is performed by the MAP3 kinase ZAKα and it promotes the activation of the downstream kinases JNK and p38, leading to cell death. Recent studies explored these models in the context of RNase L and revealed that RNase L can activate ZAKα and that loss of ZAKα results in a slower rate of apoptosis (Karasik et al. 2024; Xi et al. 2024). This finding therefore resolves the longstanding question over the mechanism of how RNase L causes cell death. Critically, our work demonstrated that activation of ZAKα depends on the catalytic activity of RNase L, pointing to the importance of RNA cleavage in stalling ribosomes. In addition, the endonuclease RNase A recapitulates the observations of RNase L, including ZAKα activation, further demonstrating that RNA fragments drive this phenomenon. This brings up the possibility that other biologically important endonucleases could induce the RSR in the same way, including IRE1. Indeed, we found that active human IRE1 appeared to promote mRNA fragment translation (Karasik et al. 2024).

These studies therefore establish a link between RNA fragmentation, activation of the RSR, and cell death (Karasik et al. 2024; Xi et al. 2024) (Figure 2). They also highlight additional potential therapeutic targets (i.e., ZAKα and downstream kinases) to inhibit RNase L's activity in cases such as autoimmune diseases (Lee et al. 2023; Magg et al. 2021). Interestingly, ZAKα can be somewhat activated by stalled ribosomes without strict need for ribosome collisions (Vind et al. 2020). Some evidence suggests that disomes become more abundant in cells during RNase L activation (Xi et al. 2024). However, further work is needed to establish how often disomes vs. stalled ribosomes are generated by RNase L activation.

Intriguingly, many processes are now known to be controlled by ribosome collisions and ZAKα activation. It is therefore conceivable that the collisions created by active RNase L could impact other pathways involved in stress adaptation or disease. For instance, both ZAKα and RNase L activation lead to inflammasome activation (Chakrabarti et al. 2015; Robinson et al. 2022) so additional work is needed to determine whether these observations relate to the same pathways. Additionally, it was recently demonstrated that inhibiting ZAKα activation prolonged lifespan and prevented obesity in mice (Snieckute et al. 2023). Whether RNase L activation is therefore involved in aging and obesity remains to be investigated (Andersen et al. 2007).

Viruses, such as vaccinia, can also induce ribosome collisions during infection (Sundaramoorthy et al. 2021). Interestingly, RNase L is triggered by vaccinia virus (Díaz‐Guerra, Rivas, and Esteban 1997), suggesting that the RSR could be promoting collisions during infection, but this needs to be further explored. Furthermore, other proteins that recognize ribosome collisions, such as ZNF598, an E3 ubiquitin ligase, have been suggested to exhibit both proviral (DiGiuseppe et al. 2018) and antiviral (Liu et al. 2023) activities. The exact functions of these ribosome collision‐related proteins during RNase L activation are therefore unclear and warrant further examination.

## 4 The Role of RNase L in Nuclear mRNA Processing and Retention

While RNase L is primarily localized in the cytoplasm, it unexpectedly also affects processes that take place in the nucleus, such as splicing, transcription termination, and nuclear export of mRNAs (Burke et al. 2021a, 2022, 2021b) (Figure 2). A proposed mechanism to explain this observation is that during RNase L activation the widespread mRNA decay frees many RNA Binding Proteins (RBPs) in the cytoplasm that translocate to the nucleus. These RBPs then are thought to alter processes that take place in the nucleus (Burke et al. 2021a, 2021b). Indeed, Burke et al. showed that several RBPs, including the polyA binding protein PABPC1, localize in the nucleus (Shown in Figure 2) and in granules termed “RNase L bodies” upon the loss of cytoplasmic RNAs during RNase L activation (reviewed in (Burke 2023) and discussed in more detail below). Interestingly, in the same study, the authors generated RNase L fused to a nuclear localization signal (NLS) and showed that it increased RNA decay in the nucleus. This nuclear activity drove RBPs out of the nucleus, further suggesting that RNA decay can influence RBP localization.

The nuclear retention of RBPs was proposed to have several outcomes. For example, many RNase‐L dependent alternative splicing events were detected and thought to be the direct consequence of nucleic acid binding by RBPs in the nucleus (Burke et al. 2022). Additionally, activation of RNase L induces reduced transcription termination on some interferon genes (also called downstream of gene transcriptional read through or DoG), such as *IFN‐β*, resulting in longer transcripts than in *RNASEL* KO cells. Active RNase L was also shown to favor nuclear retention of this extended isoform of *IFN‐β* mRNA, however the mechanism of RNA retention is unknown (Burke et al. 2021b). As noted above, viral genomes also encode nucleases, such as PA‐X of Influenza A virus and nsp1 of SARS‐CoV‐2 (Levene, Shrestha, and Gaglia 2021; Salgueiro et al. 2024). Interestingly, it was shown that overexpression of SARS‐CoV‐2's nsp1 by itself induces widespread host mRNA cleavage and nuclear retention of *IFN‐β* (Fisher et al. 2022), mirroring the outcome of RNase L activation. These changes also occur during viral infection of dengue virus that is known to trigger activation of RNase L (Burke et al. 2021a).

However, it is still not entirely clear how both the host (via RNase L) and viruses (through viral endonucleases) take advantage of these changes in the nucleus that are driven by widespread endonuclease activity. For the virus, one possibility is they benefit from nuclear retention of host innate immune mRNAs, such as *IFN‐β*. Intriguingly, it was recently shown that SARS‐CoV‐2 and MERS‐CoV (pathogenic coronaviruses) both repress the interferon response, in contrast to common cold coronaviruses that induce a more robust interferon response in the nose epithelium (Otter et al. 2024). This ability of the pathogenic coronaviruses may help explain why they can further spread in the body. Thus, these viral endonucleases may also have a role to keep interferon mRNAs in the nucleus and help evade host immune response. On the other hand, overactivation of the interferon pathways can also lead negative outcomes, and hence the host may utilize RNase L's activity to downregulate the immune response by retaining IFN‐β in the nucleus.

## 5 RNase L's Role in Cytoskeleton and Immunity

Besides being an endonuclease, RNase L was also proposed to play an additional and unusual function in the cytoskeleton. RNase L's role in the cytoskeleton was first described in cell fractionation experiments where it was observed that an insoluble form of RNase L is found in the cytoskeleton fraction and released upon its phosphorylation (Tnani, Aliau, and Bayard 1998). Later, it was shown that RNase L can directly associate with Filamin A, an actin binding protein involved in reorganization of the cytoskeleton (Malathi et al. 2014). Filamin A was reported to primarily interact with the ankyrin repeat domain of RNase L and the entry of viruses into the cell was greatly enhanced when this domain was deleted, consistent with a role for these interactions (Malathi et al. 2014). Based on this, it was proposed that monomeric RNase L is associated with the cytoskeleton and promotes the inhibition of viral entry, but once viral dsRNA is detected in the cytoplasm, RNase L is released upon dimerization to fulfill its endonucleolytic function (Malathi et al. 2014).

More recently, it was shown that replication of Zika virus relies on this cytoskeletal role of RNase L (Whelan et al. 2019, 2021). Zika virus is an RNA virus that belongs to the Flavivirus family, and it is known to strongly trigger RNase L activity (Whelan et al. 2019). Surprisingly, Zika virus replication and protein synthesis are not affected by this strong endonucleolytic activity of RNase L, and the lack of RNase L slightly decreases viral titers. This was not expected since other members of the Flavivirus family, also known to strongly trigger RNase L, tend to have increased viral titers when RNase L is not present (Whelan et al. 2019). Later it was found that RNase L promotes cytoskeleton rearrangements to enhance formation of Zika virus replication factories (Whelan et al. 2021). This observation was used to explain the disadvantage of Zika virus replication in the absence of RNase L (Figure 2) (Whelan et al. 2021). In this case, the virus has adapted to take advantage of RNase L's non‐canonical role in binding cytoskeletal proteins and unexpectedly turned it into a pro‐viral factor.

Interestingly, further direct interactions between the cytoskeleton and RNase L were also reported, but no clear functional role was associated to these interactions (Ezelle, Malathi, and Hassel 2016; Gupta and Rath 2014b; Sato et al. 2010). Whether these interactions have a role in host defense or are involved in building Zika virus replication factories remain to be answered in the future.

## 6 RNase L Induces RNase‐L Dependent Body Formation in the Cytoplasm

Another unexpected consequence of RNase L activation is formation of specific bodies in the cytoplasm (termed RNase‐L dependent bodies or RLBs) containing a unique composition of proteins and RNA that are distinct from stress granules (Burke et al. 2020) (reviewed in more detail in (Burke 2023; Watkins and Burke 2024a)). Stress granules serve as important hubs for modulating the stress response and antiviral signaling. The widespread RNA cleavage can lead to inhibition of stress granule formation while RLBs can concentrate RNase‐L degraded RNAs. A potential function of RLBs is to isolate problematic viral RNA fragments from host factors. For example, flaviviruses contain highly structured 3'UTR regions that can inhibit, and therefore sequester, the host decay machinery, such as the exonuclease, XRN1. This inhibits the decay of their RNA genome, thus increasing viral titers. Watkins et al. showed that RLBs can associate with these 3′UTR fragments of flavivirus genomes and physically separate these from XRN1, suggesting a direct role for RLBs in fighting viruses (Watkins and Burke 2024b). However, how RLBs are formed and whether RLBs can further modulate signaling, such as ZAKα activation (discussed above), remains an open question.

## 7 RNase L's Contributions to the Overall Innate Immune Response

**FIGURE 3:**
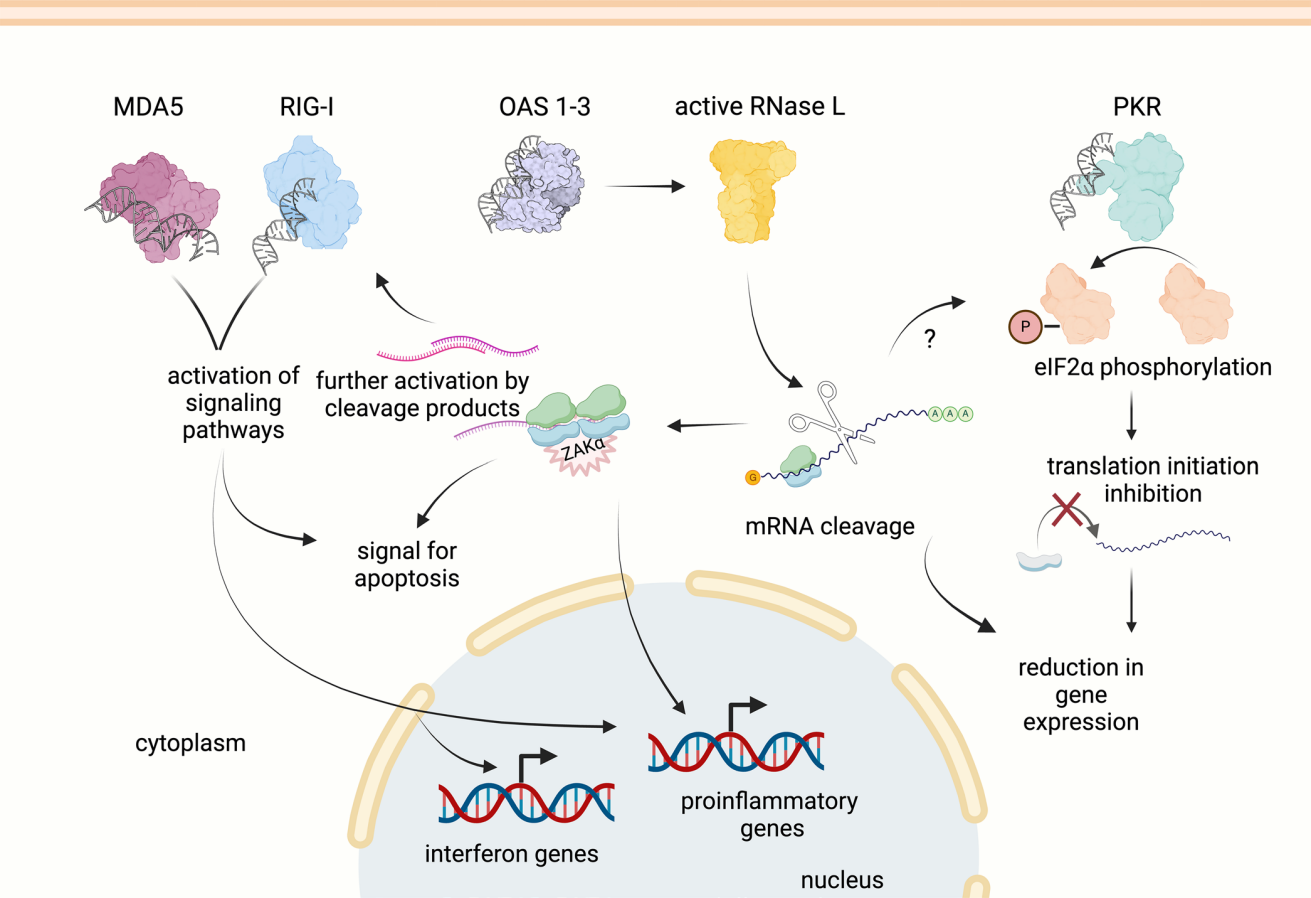
The OAS‐RNase L pathway contributes to and further amplifies the effects of activation of other dsRNA pathways, such as MDA5/RIG‐I and PKR. In particular, RNase L enhances apoptosis, promotes new transcription, and, on average, reduces the overall level of gene expression.

Besides the OAS‐RNase L and the PKR‐mediated pathways, the MDA‐5 and RIG‐I pathway can be also activated by dsRNA and this leads to the interferon and inflammatory responses (Hartmann 2017). How these different pathways act together, and the extent of OAS‐RNase L pathway's contribution during the innate immune response, is still not fully understood. Some of the functions of the OAS‐RNase L and the other dsRNA pathways are redundant, such as limiting the cell's ability to make protein and activating the inflammatory and interferon responses (Figure 3).

### 7.1 RNase L Plays a Role in Inducing Signaling and Amplifying Effects of Other dsRNA Pathways

We and other groups utilized *RNASEL KO* cells in RNA‐seq experiments to dissect the contribution of RNase L to the dsRNA response (Burke et al. 2019; Karasik et al. 2024; Rath et al. 2019). These studies found that active RNase L can induce an inflammatory response and apoptosis by itself (activation with 2–5A) and these effects serve to amplify the other dsRNA pathways (Karasik et al. 2024). More broadly, many differentially expressed transcripts were shown to rely on RNase L to be upregulated during the dsRNA‐induced innate immune response. In addition, a considerable (~50%–70%) amount of the activation of JNK and p38 MAP kinases in the dsRNA response occurs through RNase L and ZAKα, a sensor of ribosome collisions (discussed above) (Karasik et al. 2024). The other dsRNA‐mediated pathways don't activate ZAKα and thus their contributions to activating these pathways are independent from ribosome stalling and collisions. Additionally, we found that RNase L contributes ~20% toward the activation of the transcription factor IRF3, a major regulator of the interferon response. Since it was shown that RNase L‐produced RNA fragments can activate RIG‐I (Malathi et al. 2007), it is possible that this IRF3 activation is caused by RIG‐I sensing of the cleavage products. However, this model needs further testing.

Another key part of the dsRNA response is the regulation of translation by phosphorylation of the eIF2α protein (Burke et al. 2019; Karasik et al. 2024). eIF2α is required for translation initiation and its phosphorylation globally inhibits translation initiation (reviewed elsewhere (Costa‐Mattioli and Walter 2020; Wek, Jiang, and Anthony 2006)). There are four kinases that are known to phosphorylate eIF2α, including the dsRNA sensor Protein Kinase R (PKR), that is activated when dsRNA is detected in the cell (Wek, Anthony, and Staschke 2023). Interestingly, deletion of PKR does not fully abolish eIF2α phosphorylation under conditions where the dsRNA response is activated (Burke et al. 2019). Additionally, loss of RNase L alone only partially abolishes eIF2α phosphorylation (Burke et al. 2019; Karasik et al. 2024). Full elimination of phosphorylation requires the knockout of both RNase L and PKR. These data show that, in addition to PKR, another kinase is regulated by RNase L to control levels of eIF2α phosphorylation. A potential kinase that could fulfill this role is GCN2, which is activated by ribosome collisions (Meydan and Guydosh 2020; Pochopien et al. 2021; Wu et al. 2020; Yan and Zaher 2021). Since RNase L may induce ribosome collisions (see above discussion), it is conceivable that GCN2 could be activated by RNase L. However, RNase L activation in the absence of other dsRNA‐sensing pathways (i.e., via 2–5A) does not induce eIF2α phosphorylation (Karasik et al. 2021), suggesting that this sort of model would require additional regulation. An additional component of the model that is consistent with the data relies on the observation that RNase L activation leads to heavy degradation and translational repression of the mRNA that encodes GADD34 (Novoa et al. 2001), a phosphatase of eIF2α. *GADD34* transcription is only activated during the dsRNA response, potentially explaining why RNase L appears to enhance eIF2α phosphorylation when activated in the context of dsRNA but not directly by 2–5A (Karasik et al. 2024). A better understanding of RNase L's role in eIF2α phosphorylation and, more broadly, how dsRNA pathways contribute to the activation of downstream signaling and ultimately physiological changes in cells will require further study.

### 7.2 Contribution of the Different dsRNA Pathways to Aicardi‐Goutières Syndrome

ADAR1 is an adenosine‐deaminase that acts on dsRNA and promotes the separation of double‐stranded RNA to single‐stranded RNA, thus preventing the activation of dsRNA sensors in normal cells (reviewed here (Song et al. 2022)). Mutations in ADAR1 result in Aicardi‐Goutières syndrome (AGS), causing interferonopathy, which is the overproduction of interferons and overactivation of ISGs (Rice et al. 2012). Interestingly, ADAR1 deficiency can be alleviated by individually blocking the major cytoplasmic dsRNA pathways (discussed above), including OAS/RNase L (Bajad et al. 2020; Daou et al. 2020; Li et al. 2017; Maurano et al. 2021). For example, some ADAR1 patient mutations activate RNase L and this leads to cell death in cell culture. This outcome can be rescued by RNase L inhibitors or by knocking out the *RNASEL* gene, consistent with a major role for RNase L in the disease (Daou et al. 2020; Li et al. 2017). In another example, deletion of MAVS (Mitochondrial Antiviral Signaling Protein), a protein that plays a role in triggering the interferon response by acting as an intermediate signaling factor downstream of the dsRNA sensors RIG‐I and MDA5, can also rescue some of the ADAR1 mutations in vivo (Bajad et al. 2020). ISRIB, an inhibitor of the Integrated Stress Response and the effects of PKR‐triggered eIF2α phosphorylation, was also able to counteract AGS disease pathogenesis in mice (Maurano et al. 2021). This suggests that each of these dsRNA pathways, in addition to the OAS‐RNase L axis, contribute to the disease and the most severe disease outcomes may be consequences of their concerted actions. However, the exact contributions of each dsRNA pathway in the development of ADAR1 mutation induced AGS remain to be elucidated in the future. Dissecting the role of these pathways may lead to the development of therapeutic approaches targeting more than one of the dsRNA pathways simultaneously.

## 8 Recent Discoveries Link the Unusual Roles of RNase L to Disease

**FIGURE 4:**
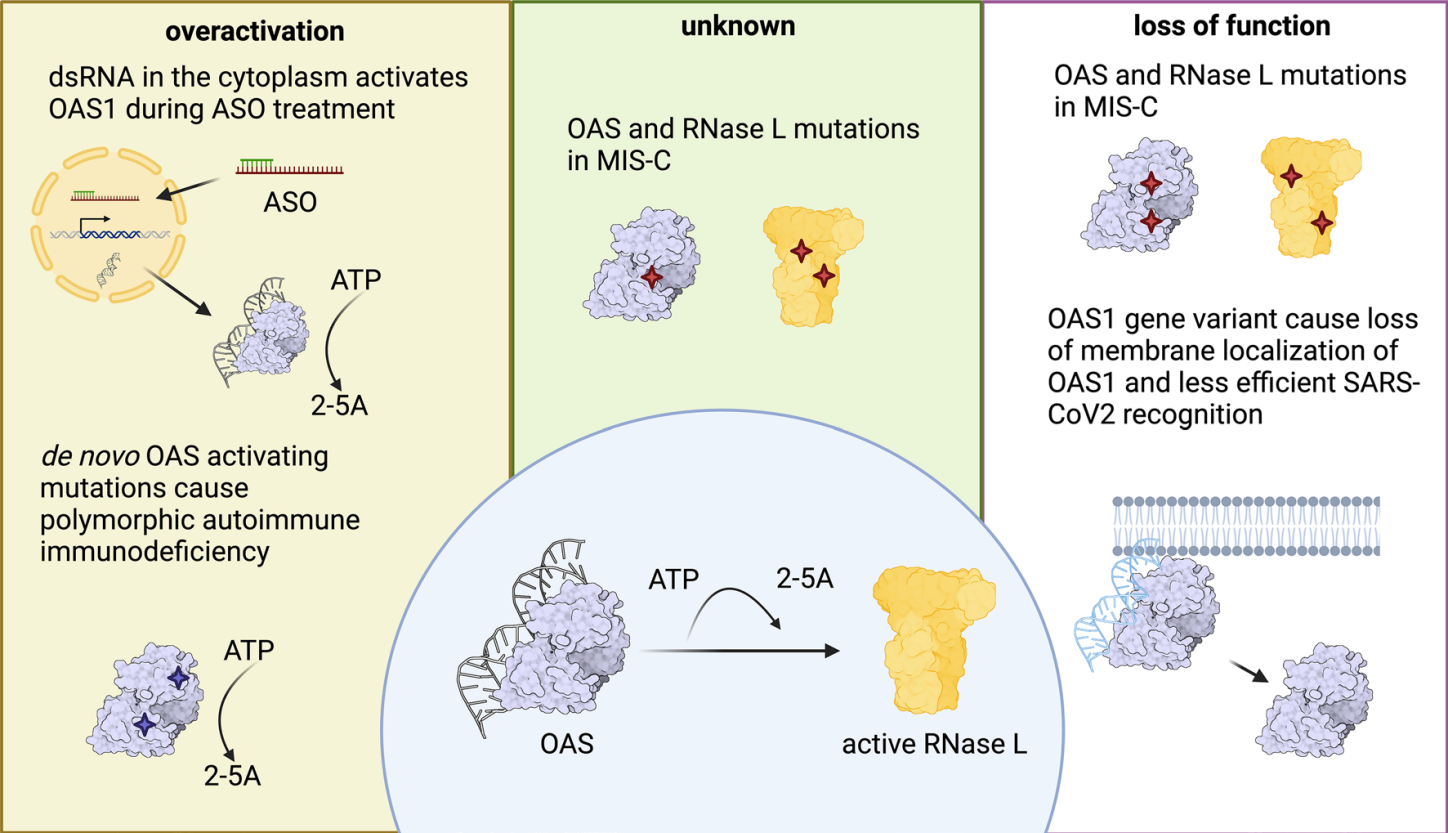
The role of OAS‐RNase L pathway in recently characterized human diseases or disease therapies.

RNase L activation is implicated in many human diseases which, in many cases, uncovered further surprising roles of this broadly‐acting endonuclease (Figure 4). RNase L can be activated by a large subset of viruses, including Flaviviruses (e.g., Zika, West Nile virus), SARS‐CoV‐2, and Influenza A (reviewed in (Silverman 2007)). Besides RNase L's antiviral function, it is also thought to be an anticancer factor (reviewed here in detail (Bisbal and Silverman 2007; Prangley and Korennykh 2022)). This is supported by the finding that RNase L mutations increase risk for multiple types of cancer, including Hereditary Prostate cancer and breast cancer (Casey et al. 2002; Madsen et al. 2008). Furthermore, the activation of RNase L is also connected to autoimmune disease (Cho et al. 2018; Magg et al. 2021) (reviewed below). Here we summarize the most recent discoveries on the role of RNase L in human health and disease.

### 8.1 Roles of RNase L Activation Related to SARS‐CoV‐2 Infection

As the SARS‐CoV‐2 pandemic emerged, it became clear that loss of function in the OAS‐RNase L pathway plays a significant role in pathogenesis of the disease. Genetic differences in OAS genes were linked to higher risk of hospitalizations in patients infected with SARS‐CoV‐2 (Banday et al. 2022). For example, one of the single‐nucleotide polymorphisms associated with the OAS1 gene (SNP Rs10774671) modulates the expression of membrane‐bound and cytosolic forms of OAS1 (Wickenhagen et al. 2021). Membrane‐bound OAS1 is more efficient in recognizing SARS‐CoV‐2 RNA than cytosolic OAS1 and thus plays a role in activating RNase L during infection by this virus (Figure 4) (Wickenhagen et al. 2021). Additionally, loss of function mutations in OASs (OAS1‐2) and RNase L were also linked to Multisystem Inflammatory Syndrome in Children (MIS‐C), an autoimmune disease that occurs weeks after SARS‐CoV‐2 infection in some children (Lee et al. 2023). These mutations were proposed to be the underlying cause of MIS‐C. Interestingly, two of the RNase L disease‐causing mutations, G59S and E265*, were previously indicated in prostate and breast cancers, suggesting that patients with these mutations are susceptible to multiple diseases (Nguyen‐Dumont et al. 2018; Rökman et al. 2002). MIS‐C patients with mutations in the OAS‐RNase L pathway typically have an asymptomatic or mild infection in the first few weeks, an indication that the OAS‐RNase L pathway may not have a significant role in the initial viral infection. Then, 2–4 weeks after the development of the viral infection, and after it has cleared, MIS‐C symptoms arise due to an overactivation of the immune response. Thus, RNase L may have a role in attenuating this response, preventing overactivation and development of the autoimmune disease. Intriguingly, we also observed that some interferon‐stimulated genes (e.g., *IFIT3*) are regulated by RNase L during activation in cells (Karasik et al. 2024), further supporting the idea that RNase L has an inhibitory role in the immune response. While RNase L is thought to be pro‐inflammatory, this additional and unexpected role in repressing innate immune genes suggests that OAS‐RNase L may have opposing activities at different stages of an infection.

### 8.2 RNase L Is Linked to Autoinflammation

Loss‐of‐function mutations in the RNase L gene have been linked to the development Inflammatory Bowel Disease (IBD) in patients (Kim 2019) and lack of RNase L in mice was found to exacerbate colitis, a form of IBD (Long et al. 2013). Recently, it was described that overactivation of the OAS‐RNase L pathway can also lead to pathogenesis that includes symptoms of IBD. Novel OAS1 patient mutations were linked to hyperactivation of RNase L, leading to polymorphic autoinflammatory immunodeficiency, a disease characterized by several symptoms, including those of IBD (Magg et al. 2021) (Figure 4). Inhibition of RNase L by curcumin, a non‐competitive inhibitor of RNase L (Gupta and Rath 2014a), rescues cells from the effects of RNase L activation—a promising approach for therapies for these patients. These findings further highlight the importance of RNase L in this autoimmune disease and suggest a potentially homeostatic role for the OAS/RNase L pathway where achieving appropriate level of activation is critical.

### 8.3 RNase L Is Activated by Antisense Oligonucleotides

Nucleic acid (DNA or RNA) therapeutics are on the rise (reviewed here (Belgrad, Fakih, and Khvorova 2024)). Therefore, it is important to understand their effects on the dsRNA response to avoid or mitigate any side effects and understand the associated risks of treatment. Interestingly, OAS and RNase L activation was observed during Antisense Oligonucleotide (ASO) treatment (Chitrakar et al. 2021). ASOs are short single‐stranded oligonucleotides that are used to treat a number of diseases, such as Spinal Muscular Atrophy. Chitrakar et al. found that during ASO treatment the decay of nuclear RNAs was inhibited and that resulted in an increase of endogenously expressed dsRNA (intergenic inverted retroelements) in the nucleus. This increased dsRNA in the nucleus was proposed to spill over to the cytoplasm where it activated the OAS‐RNase L pathway (Figure 4). Surprisingly, these dsRNAs from the nucleus did not trigger the production of interferons but did activate RNase L, suggesting a narrow spectrum of dsRNA sensors that were able to detect them. This study highlights the need for evaluating these ASO drugs in the context of dsRNA production and OAS‐RNase L activation.

## 9 Conclusions and Future Directions

Recent discoveries have revealed and improved our understanding of the molecular mechanisms underlying the unusual roles of RNase L activation. In particular, RNase L's ability to induce the translation of RNA fragments and interfere with nuclear RNA export have revealed novel ways that endonucleolytic cleavage of the host's mRNA produce important physiological outcomes for the cell.

However, many aspects of RNase L activation remain to be investigated. RNase L was shown to induce different cellular processes, particularly apoptosis and autophagy. How these distinct processes are coordinated and whether ribosome collisions play a more sophisticated role in them needs to be addressed in the future. Another exciting area is the further development of RNase L activators and inhibitors for therapeutic use (Haniff et al. 2020; Lášek et al. 2022; Su et al. 2021; Thakur et al. 2007; Tong et al. 2023), however, these still need to be tested in the clinic. Furthermore, it is intriguing that other non‐specific RNases can recapitulate effects of RNase L activation, including viral endonucleases, such as nsp1. These unusual aspects of generic RNases could be harnessed for developing therapeutics. In fact, RNases are already used or are being further developed for cancer therapies (Siraj 2022) (e.g., Onconase is an FDA approved RNase). Understanding the molecular mechanism and the complex network of downstream effects could open new ways to approach therapies.

## Author Contributions

**Agnes Karasik:** conceptualization (lead), visualization (lead), writing – original draft (lead), writing – review and editing (equal). **Nicholas R. Guydosh:** conceptualization (supporting), funding acquisition (lead), supervision (lead), writing – review and editing (equal).

## Conflicts of Interest

The authors declare no conflicts of interest.

## Related WIREs Articles

Regulation of ribonucleoprotein condensates by RNase L during viral infection.

## Data Availability

Data sharing is not applicable to this article as no new data were created or analyzed in this study.
